# Prevalence and severity of anxiety, stress, and depression among adults with long COVID in Barcelona: a cross-sectional study

**DOI:** 10.3399/BJGPO.2024.0098

**Published:** 2025-07-16

**Authors:** Brenda Biaani León-Gómez, Meritxell Carmona-Cervelló, Rosalia Dacosta-Aguayo, Noemí Lamonja-Vicente, Jofre Bielsa-Pascual, Victor M López-Lifante, Valeria Zamora-Putin, Gemma Molist, Pilar Montero-Alia, Alba Pachón-Camacho, Eduard Moreno-Gabriel, Rosa García-Sierra, Adrià Bermudo-Gallaguet, Carla Chacón, Anna Costa-Garrido, Jose A Muñoz-Moreno, Lourdes Mateu, Maria Mataró, Julia G Prado, Eva Martínez-Cáceres, Marta Massanella, Concepción Violán, Pere Torán-Monserrat, Aitana Ayet, Sandra Banderas, Laia Bernard, Jofre Bielsa-Pascual, Marc Boigues, Meritxell Carmona-Cervelló, Lucía A Carrasco-Ribelles, Carla Chacón, Anna Costa-Garrido, Galadriel Diez Fadrique, Rosalía Dacosta-Aguayo, Antònia Fornés, Rosa García-Sierra, Eulàlia Grau, Noemí Lamonja-Vicente, Brenda B León-Gómez, Liudmila Liutsko, Gemma Lladós, Cristina López, Víctor M López-Linfante, Cora Loste, Marta Massanella, Maria Mataró, Lourdes Mateu, Eva M Martínez-Cáceres, Gemma Molist, Pilar Montero-Alia, Eduard Moreno-Gabriel, Francisco Muñoz-López, Jose A Muñoz-Moreno, Maria Nevot, Alba Pachón, Ruth Peña, Raul Pérez-Caballero, Julia G Prado, Anna Prats, Josep Puig, Bibiana Quirant, Gabriel F Rodriguez-Lozano, M Carmen Rodríguez-Pérez, Sandra Sánchez-Vallespín, Jose Ramón Santos, Pere Torán-Monserrat, Macedonia Trigueros, Concepció Violán, Valeria Zamora-Putin

**Affiliations:** 1 Unitat de Suport a la Recerca Metropolitana Nord, Institut Universitari d’Investigació en Atenció Primària Jordi Gol (IDIAP Jordi Gol), Mataró, Spain; 2 Germans Trias i Pujol Research Institute (IGTP), Badalona, Spain; 3 Grup de Recerca en Impacte de les Malalties Cròniques i les seves Trajectòries (GRIMTra)2021 SGR 01537, Barcelona, Institut Universitari d’Investigació en Atenció Primària Jordi Gol (IDIAPJGol), Spain; 4 Multidisciplinary Research Group in Health and Society(GREMSAS), Institut Universitari d’Investigació en Atenció Primària Jordi Gol (IDIAPJGol), Barcelona, Spain; 5 Palau-Solità Healthcare Centre, Palau-Solità Plegamans Institut Català de la Salut, Barcelona, Spain; 6 Department of Medicine, Universitat Autònoma de Barcelona, Bellaterra, Spain; 7 Faculty of Medicine, University of Vic-Central University of Vic (UVic-UCC), Catalonia, Vic, Spain; 8 Department of Social Psychology, Universitat Autònoma de Barcelona, Cerdanyola de Vallès, Bellaterra, Spain; 9 Nursing Department, Faculty of Medicine, Universitat Autònoma de Barcelona, Barcelona, Spain; 10 Department of Clinical Psychology and Psychobiology, University of Barcelona, Barcelona, Spain; 11 Infectious Diseases Department, Fundació Lluita contra les infeccions, Germans Trias i Pujol Hospital, Badalona, Spain; 12 Faculty of Psychology and Education Sciences, Universitat Oberta de Catalunya (UOC), Barcelona, Spain; 13 Centro de Investigación Biomédica en Red de Enfermedades Infecciosas (CIBERINFEC), Instituto de Salud Carlos III (ISCIII), Madrid, Spain; 14 Red Española de investigación en Covid Persisitente (REiCOP), Barcelona, Spain; 15 Institut de Neurociències, University of Barcelona, Barcelona, Spain; 16 Institut de Recerca Sant Joan de Déu, Esplugues de Llobregat, Spain; 17 IrsiCaixa-AIDS Research Institute, Can Ruti Campus, Badalona, Spain; 18 Immunology Department, FOCIS Center of Excellence, Universitat Autònoma de Barcelona, Bellaterra, Cerdanyola del Vallès, Spain; 19 Immunology Division, Laboratori Clínic Metropolitana Nord (LCMN), Hospital Universitari Germans Trias i Pujol, Badalona, Spain; 20 Red de Investigación en Cronicidad, Atención Primaria y Prevención y Promoción de la Salud (RICAPPS). Instituto de Salud Carlos III (ISCIII), Madrid, Spain; 21 Department of Medicine, Faculty of Medicine, Universitat de Girona, Girona, Spain

**Keywords:** long COVID, post-COVID-19 condition, post-acute COVID-19 syndrome, mental health, primary health care, general practitioners

## Abstract

**Background:**

The COVID-19 pandemic’s long-term mental health implications are increasingly concerning, especially among patients with post-acute sequelae of SARS-CoV-2 infection, also known as long COVID (LC).

**Aim:**

To explore the presence and distribution of anxiety, depression, and stress in individuals with LC with cognitive complaints in northern Barcelona, Spain.

**Design & setting:**

This cross-sectional study involved 155 diagnosed individuals with LC from the ’Aliança ProHEpiC-19 Cognitiu (APC)’ project.

**Method:**

Demographic data and health behaviour variables were collected, and the Depression, Anxiety, and Stress Scale (DASS-21) was self-administered to assess mental health. Descriptive statistics, χ^2^ tests, and Poisson regression models were used for data analysis.

**Results:**

’Severe’ stress and ’extremely severe’ anxiety were prevalent in the sample. There were significant differences in anxiety and depression based on age and job role, with older individuals and non-healthcare workers showing higher relative risks (*P*<0.05).

**Conclusion:**

Our study highlights the significant mental health burden in patients with LC, underscoring the need for targeted interventions, especially among adults aged >45 years and non-healthcare workers. Further research is needed to better understand LC’s complex mental health impacts and develop effective clinical management strategies.

## How this fits in

Some individuals infected with SARS-CoV-2 have presented with a variety of mid- to long-term symptoms, which have been described as long COVID (LC). This article explores the presence and distribution of symptoms of anxiety, depression, and stress in individuals with LC and cognitive complaints. High levels of stress, anxiety, and depression were observed across sex categories, work settings, and educational levels. The results of this study underscore the necessity of addressing mental health concerns in patients with LC. It is therefore recommended that emotional and psychological support be incorporated into clinical management plans.

## Introduction

Long COVID (LC) is defined as the continuation or development of new symptoms 3 months after the initial SARS-CoV-2 infection, with these symptoms lasting for at least 2 months with no other explanation.^1^ These symptoms, which can include fatigue, breathlessness, cognitive dysfunction, and psychological effects, may persist, come and go, or relapse over time and could affect a person’s ability to perform daily activities. These individuals may refer to themselves as ’long-haulers’.^2^

LC is linked to mental health issues, such as anxiety, depression, and stress, with degrees of severity.^3,4^ Some neuropsychological symptoms are prevalent during both the acute and chronic phases of COVID-19.^5^ Commonly reported mental health problems include anxiety (6.5–63%), depression (4–31%), and post-traumatic stress disorder (12.1–46.9%).^6^ Epidemiological estimates for depressive symptoms in LC vary, affecting 14–52% of discharged patients,^5,7^ and approximately 35% of patients in short to long-term follow-ups.^8^ The psychiatric impact of COVID-19 is mainly related to the immune system’s inflammatory response, potentially causing neuroinflammation and psychological distress owing to SARS-CoV-2.^9–12^

Knowing the neuropsychological conditions (stress, anxiety, and depression) of individuals with LC is essential to help healthcare providers understand how these conditions may interact with the virus and exacerbate its effects, thus contributing to a holistic management of long-term COVID-19.^3,4^ This approach could encompass the identification of risk factors and overlapping symptoms, as well as the implementation of treatments designed to address multiple conditions simultaneously. Conducting a local survey in Barcelona, Spain, is particularly necessary owing to unique regional health dynamics and socio-cultural factors that may influence the presentation and management of LC. Understanding these local nuances is crucial as they provide insights that are relevant to wider populations by highlighting how localised data can inform broader healthcare strategies. Moreover, this information is highly relevant to general practice as it equips healthcare providers with specific knowledge about the mental health impacts of LC in their community, allowing for more personalised and effective patient care. In addition, this information could be key to making therapeutic decisions and guiding research towards the development of better treatments for those with comorbidities. Describing the status of individuals with LC can help identify populations at a higher risk, which can aid in developing targeted interventions and education campaigns to support these populations. In this study, we aim to analyse the presence and distribution of anxiety, depression, and stress in patients presenting with LC with cognitive complaints in northern Barcelona (Spain).

## Method

### Source of information

The Aliança ProHEpiC-19 Cognitiu (APC) study recruited volunteer participants (healthcare and non-healthcare workers) from primary care or hospital care centres from the public network northern Barcelona (Spain) from 1 August 2020–March 2023. Further details of the APC project can be found in the study protocol.^13^ This cross-sectional study includes a subset of diagnosed patients with LC (155 patients) from the APC project. The APC project used the data from patients aged between 25 years and 70 years with post- and non-LC conditions. All the patients in this subset were diagnosed with LC, defined as the latest World Health Organization (WHO) definition.^1^ Participants with previous established diagnosis of psychiatric, neurological, or neurodevelopmental disorders that caused cognitive deficits before COVID-19 infection were excluded from the sample. In addition, patients with a history of drug or alcohol abuse were excluded.

### Collection and description of the variables

Data collection was carried out in successive steps. First, through face-to-face surveys, researchers collected the following sociodemographic information: age (<35 years; 35–44 years; 45–54 years; and 55–70 years); sex and gender (the categories of male, female, and other genders were considered. However, there were no participants who identified within the ’other’ category, so the analyses were limited to the male and female categories only); education (non-university, university); and job field (doctor, nurse, health services, health assistant, and social worker and ’others’ [all professions not related to health and social care]). Health behaviour variables addressed were tobacco (never smoked, currently smokes, used to smoke); alcohol consumption (yes or no); anthropometric parameters (body mass index [BMI], according to the WHO classification); and comorbidities (hypertension, cholesterol, and diabetes). Moreover, they were also asked about their COVID-19 experience (time of disease onset, diagnostic method, and clinical spectrum including symptoms and treatment).

Afterwards, participants answered the validated questionnaire of Depression, Anxiety, and Stress Scale 21 (DASS-21) to assess mental health variables.^14^ The DASS-21 includes 21 items across three self-reported subscale scores of depression, anxiety, and tension or stress. Each of the seven elements on the subscales (depression, anxiety, and tension or stress) are scored on a Likert scale from 0–3 (0 = ’Did not apply to me at all’; 1 = ’Applied to me to some degree or some of the time’; 2 = ’Applied to me to a considerable degree or a good part of time’; 3 = ’Applied to me very much or most of the time’). Depression was classified as normal (≤4), mild (5–6), moderate (7–10), severe (11–13), and extremely severe (≥14). Anxiety was classified as normal (<4), mild (4), moderate (5–7), severe (8–9), and extremely severe (≥10). Stress was classified as normal (<8), mild (8–9), moderate (10–12), severe (13–16), and extremely severe (≥17). The maximum score for the DASS-21 was 63, and the questionnaire took about 5–10 minutes to complete.

### Statistical analysis

All analyses were stratified by sex. Descriptive proportions and frequencies were calculated for four age groups, job, educational level, hypertension, cholesterol, diabetes, alcohol use, smoking status, and BMI. Pearson’s χ^2^ tests were used to evaluate differences in stress, anxiety, and depression among stratified groups. Poisson regression models with robust variance were fitted to obtain the prevalence ratio of stress, depression, and anxiety (crude model 1) for sex (model 2), educational level (model 3), and job field (model 4) with men, lower educational level, and non-healthcare workers as the reference. These analyses were performed using Stata (version 14).

## Results

Total sample size was 155, with a sex distribution of 126 females (81.30%) and 29 males (18.70%). The largest proportion of females (46.03%) and males (55.17%) were in the age groups of 45–54 years and ≥55 years, respectively. The majority worked in ’other’ fields (60.00%), with more males (79.31%) than females (55.56%). No significant differences were found in educational level between sexes. Hypertension, elevated cholesterol, and diabetes were more common in males than females. Alcohol consumption was higher in males (51.72% against 32.00%), while smoking habits and BMI were similar between sexes (Table 1).

**Table 1.** Patients with long COVID (*n* = 155) by sex among job field, educational level, hypertension, cholesterol, diabetes, alcohol, smoking, body mass index

	Female	Male		Total
**Variables**	*n* (%)	*n* (%)	*P*	*n* (%)
**Age, years**				
20–34	7 (5.56)	0 (0.00)	0.004^a^	7 (4.52)
35–44	33 (26.19)	5 (17.24)		38 (24.52)
45–54	58 (46.03)	8 (27.59)		66 (42.58)
55–70	28 (22.22)	16 (55.17)		44 (28.39)
**Job field**				
Doctor	7 (5.56)	2 (6.90)	0.129	9 (5.81)
Nurse	23 (18.25)	4 (13.79)		27 (17.42)
Services	10 (7.94)	0 (0.00)		10 (6.45)
Assistant	15 (11.90)	0 (0.00)		15 (9.68)
Social worker	1 (0.79)	0 (0.00)		1 (0.65)
Other	70 (55.56)	23 (79.31)		93 (60.00)
**Educational level**				
Non-university	58 (46.03)	18 (62.07)	0.119	76 (49.03)
University	68 (53.97)	11 (37.93)		79 (50.97)
**Hypertension**				
No	108 (85.71)	14 (48.28)	0.000^a^	122 (78.71)
Yes	18 (14.29)	15 (51.72)		33 (21.29)
**High cholesterol**				
No	101 (80.16)	18 (62.07)	0.038^a^	119 (76.77)
Yes	25 (19.84)	11 (37.93)		36 (23.23)
**Diabetes**				
No	124 (98.41)	26 (89.66)	0.016	150 (96.77)
Yes	2 (1.59)	3 (10.34)		5 (3.23)
**Alcohol**				
No	85 (68.00)	14 (48.28)	0.046	99 (64.29)
Yes	40 (32.00)	15 (51.72)		55 (35.71)
**Smoking status**				
Never	74 (58.73)	12 (42.86)	0.273	86 (55.84)
Currently smokes	9 (7.14)	2 (7.14)		11 (7.14)
Used to smoke	43 (34.13)	14 (50.00)		57 (37.01)
				
**Body mass index^b^**				
Underweight	6 (4.80)	0 (0.00)	0.103	6 (3.92)
Normal weight	47 (37.60)	5 (17.86)		52 (33.99)
Overweight	39 (31.20)	12 (42.86)		51 (33.33)
Obesity class I	16 (12.80)	6 (21.43)		22 (14.38)
Obesity class II	13 (10.40)	2 (7.14)		15 (9.80)
Obesity class III	4 (3.20)	3 (10.71)		7 (4.58)

^a^ *P*<0.05. ^b^According to World Health Organization standards.

No significant differences in stress were observed based on sex, education, or job (Table 2). ’Extremely severe’ anxiety was common across both sexes and educational levels. Depression showed no significant differences by sex or education but differed between healthcare and non-healthcare workers, with ’normal’ levels more frequent in the former. The Poisson regression analysis did not identify statistically significant associations of stress with sex, age, education, or job (Table 3). Anxiety showed significant associations with age and job (*P*<0.05), being higher in older age groups and non-healthcare workers (Table 4). Depression was also significantly associated with age and job (*P*<0.05), with higher risks in older groups and non-healthcare workers (Table 5).

**Table 2.** χ^2^ analysis of DASS-21 mental health indicators among patients with long COVID: differences by sex, education, and healthcare workers

	Sex			Educational level			Healthcare workers		
	Female	Male		Non-university	University		Non-healthcare	Healthcare	
**Variables**	*n* (%)	*n* (%)	*P*	*n (%)*	*n* (%)	*P*	*n* (%)	*n* (%)	*P*
**Stress**									
Normal	20 (15.87)	3 (10.34)	0.399	6 (7.89)	17 (21.52)	0.131	12 (12.77)	11 (18.03)	0.274
Mild	20 (15.87)	4 (13.79)		15 (19.74)	9 (11.39)		13 (13.83)	11 (18.03)	
Moderate	27 (21.43)	5 (17.24)		16 (21.05)	16 (20.25)		19 (20.21)	13 (21.31)	
Severe	37 (29.37)	14 (48.28)		25 (32.89)	26 (32.91)		30 (31.91)	21 (34.43)	
Extremely severe	22 (17.46)	3 (10.34)		14 (18.42)	11 (13.92)		20 (21.28)	5 (8.20)	
**Anxiety**									
Normal	23 (18.25)	4 (13.79)	0.515	9 (11.84)	18 (22.78)	0.093	12 (12.77)	15 (24.59)	0.077
Mild	5 (3.97)	1 (3.45)		2 (2.63)	4 (5.06)		3 (3.19)	3 (4.92)	
Moderate	26 (20.63)	3 (10.34)		15 (19.74)	14 (17.72)		17 (18.09)	12 (19.67)	
Severe	16 (12.70)	3 (10.34)		14 (18.42)	5 (6.33)		9 (9.57)	10 (16.39)	
Extremely severe	56 (44.44)	18 (62.07)		36 (47.37)	38 (48.10)		53 (56.38)	21 (34.43)	
**Depression**									
Normal	29 (23.02)	6 (20.69)	0.592	12 (15.79)	23 (29.11)	0.356	16 (17.02)	19 (31.15)	0.044
Mild	20 (15.87)	3 (10.34)		11 (14.47)	12 (15.19)		12 (12.77)	11 (18.03)	
Moderate	28 (22.22)	5 (17.24)		18 (23.68)	15 (18.99)		19 (20.21)	14 (22.95)	
Severe	16 (12.70)	3 (10.34)		11 (14.47)	8 (10.13)		16 (17.02)	3 (4.92)	
Extremely severe	33 (26.19)	12 (41.38)		24 (31.58)	21 (26.58)		31 (32.98)	14 (22.95)	

The percentage of females is 81.30% and males is 18.70%. The proportion of non-university graduates is 49.03%, while university graduates account for 50.97%. Additionally, 60.00% are not employed in the healthcare sector, and 40.00% are employed within the healthcare sector.

**Table 3.** Poisson analysis of associations between stress levels and sex, age, education, and healthcare workers in patients with long COVID

	Model 1	Model 2	Model 3	Model 4
**Variables**	IRR with 95% CI	IRR with 95% CI	IRR with 95% CI	IRR with 95% CI
**Sex**				
Male	1	1	1	1
Female	0.96 (0.84 to 1.09)	0.99 (0.87 to 1.13)	0.99 (0.87 to 1.14)	1.02 (0.89 to 1.17)
**Age, years**				
25–34	1	1	1	1
35–44	1.22 (0.87 to 1.71)	1.22 (0.87 to 1.71)	1.22 (0.87 to 1.70)	1.22 (0.84 to 1.75)
45–54	1.33 (0.96 to 1.85)	1.33 (0.96 to 1.85)	1.32 (0.95 to 1.83)	1.33 (0.92 to 1.90)
55–70	1.39 (0.99 to 1.94)	1.38 (0.98 to 1.95)	1.38 (0.98 to 1.93)	1.41 (0.97 to 2.04)
**Education level**				
University	1	N/A	1	1
Non-university	1.05 (0.93 to 1.17)	N/A	1.02 (0.91 to 1.14)	0.99 (0.88 to 1.12)
**Healthcare workers**				
Non-health workers	1	N/A	N/A	1
Health workers	0.90 (0.79 to 1.01)	N/A	N/A	0.89 (0.78 to 1.01)

Significance was set as *P*<0.05. The Poisson regression analysis did not identify statistically significant associations of stress with sex, age, education or job. Model 1: Independent crude association model of sex, age, education and healthcare workers with stress. Model 2: model of sex+age association with stress. Model 3: model of sex+age+education association with stress. Model 4: model of sex+age+education+healthcare workers association with stress. IRR = incidence rate ratio

**Table 4.** Poisson analysis of associations between anxiety levels and sex, age, education, and healthcare workers in patients with long COVID

	Model 1	Model 2	Model 3	Model 4
**Variables**	IRR with 95% CI	IRR with 95% CI	IRR with 95% CI	IRR with 95% CI
**Sex**				
Male	1	1	1	1
Female	0.86 (0.69 to 1.05)	0.90 (0.73 to 1.12)	0.91 (0.73 to 1.12)	0.98 (0.79 to 1.20)
**Age, years**				
25–34	1	1	1	1
35–44	1.70 (1.01 to 2.87)^a^	1.68 (0.99 to 2.84)	1.67 (0.98 to 2.86)	1.67 (0.95 to 2.94)
45– 54	1.89 (1.13 to 3.15)^a^	1.87 (1.12 to 3.12)^a^	1.85 (1.10 to 3.12)^a^	1.86 (1.07 to 3.23)^a^
55–70	2.04 (1.22 to 3.41)^a^	1.97 (1.17 to 3.32)^a^	1.95 (1.14 to 3.31)^a^	2.07 (1.17 to 3.65)^a^
**Education level**				
University	1	N/A	1	1
Non-university	1.09 (0.91 to 1.31)	N/A	1.04 (0.87 to 1.25)	0.98 (0.82 to 1.17)
**Healthcare workers**				
Non-health workers	1	N/A	N/A	1
Health workers	0.75 (0.61 to 0.91)^a^	N/A	N/A	0.74 (0.60 to 0.91)^a^

^a^ *P*<0.05. Model 1: independent crude association model of sex, age, education, and healthcare workers with anxiety. Model 2: model of sex+age association with anxiety. Model 3: model of sex+age+education association with anxiety. Model 4: model of sex+age+education+healthcare workers association with anxiety. IRR = incidence rate ratio

**Table 5.** Poisson analysis of associations between depression levels and sex, age, education, and healthcare workers in patients with long COVID

	Model 1	Model 2	Model 3	Model 4
**Variables**	IRR with 95% CI	IRR with 95% CI	IRR with 95% CI	IRR with 95% CI
**Sex**				
Male	1	1	1	1
Female	0.88 (0.70 to 1.11)	0.92 (0.74 to 1.14)	0.93 (0.74 to 1.15)	0.99 (0.79 to 1.25)
**Age**				
25–34	1	1	1	1
35–44	1.34 (0.82 to 2.18)	1.32 (0.81 to 2.16)	1.32 (0.81 to 2.13)	1.31 (0.81 to 2.11)
45–54	1.60 (1.02 to 2.51)^a^	1.59 (1.01, 2.50)^a^	1.55 (0.99 to 2.44)	1.56 (1.01 to 2.42)^a^
55–70	1.65 (1.04 to 2.61)^a^	1.60 (1.00 to 2.55)^a^	1.56 (0.98 to 2.49)	1.65 (1.06 to 2.59)^a^
**Education level**				
University	1	N/A	1	1
Non-university	1.13 (0.94 to 1.37)	N/A	1.09 (0.90 to 1.31)	1.02 (0.85 to 1.23)
**Healthcare workers**				
Non-health workers	1	N/A	N/A	1
Health workers	0.76 (0.62 to 0.93)^a^	N/A	N/A	0.76 (0.62 to 0.93)^a^

^a^ *P*<0.05. Model 1: independent crude association model of sex, age, education and healthcare workers with depression. Model 2: model of sex+age association with depression. Model 3: model of sex+age+education association with depression. Model 4: model of sex+age+education+healthcare workers association with depression. IRR = incidence rate ratio

## Discussion

### Summary

The majority of the population in our sample were female. However, no significant sex differences were observed in the distribution of anxiety, stress, and depression. A substantial proportion of patients with LC also have diseases such as hypertension and high cholesterol. Furthermore, our findings highlight the widespread presence of high levels of stress, anxiety, and depression across sexes, occupational settings, and educational levels.

### Strengths and limitations

The study’s strengths include extensive follow-up and the exploration of mental health variables in a population with a newly established disease. Additionally, a validated and adapted scale has been utilised to assess the self-reported symptomatology of anxiety, stress, and depression. This study provides specific insights into the prevalence and severity of anxiety, stress, and depression among patients with LC in a specific population and across various age groups and occupational roles. This localised perspective is valuable as it can inform region-specific healthcare policies and interventions. However, the study’s limitations include a small sample size, which may limit the generalisability of the findings. Furthermore, the lack of a control group makes it challenging to definitively attribute the observed effects to LC specifically. It is possible that the study does not account for all potential confounding factors, such as pre-existing health conditions, socioeconomic status, and so forth. Furthermore, the sex imbalance observed in the study population may influence the distribution of mental health outcomes.

In addition, potential subgroups need further research according to inequality axes such as social class, ethnicity, migration, and others. Also, further research is needed to address vulnerable populations to have more information to implement targeted interventions. For instance, our study focused on binary gender categories (male and female) owing to the available data of our sample. Future studies could benefit from including a more diverse range of gender identities to provide a more comprehensive analysis. However, the results reflect the commonly observed trend of higher female participation, and we therefore recommend that future studies aim for a balanced sample with respect to sex and gender. Classifying educational attainment using a binary system of ’university’ and ’non-university’ may oversimplify the broad spectrum of educational experiences. Factors such as accessibility, affordability, and cultural attitudes towards education can be related to education.

### Comparison with existing literature

Numerous studies have suggested that females may have a higher risk of developing LC;^15,16^ this heightened vulnerability is attributed to a combination of factors. Both biological elements, including hormones and immune responses, and socio-cultural aspects, such as health-related behaviors and gender roles, contribute significantly to gender disparities in LC.^15,17^ The specific mechanisms driving the elevated risk of LC in females remain largely unexplored and require further research. In our study, we did not observe significant sex differences in the distribution of mental health conditions such as anxiety, stress, and depression. This suggests that the mental health burden is equally distributed across sexes in our sample, irrespective of the greater prevalence of LC in females. Also, there is a notable disparity in age distribution between the sexes. A substantial proportion of participants, particularly females, were between the ages of 45 years and 54 years. On the other hand, most male participants were aged ≥55 years. This age-related difference may considerably affect risk levels for stress, anxiety, and depression.

A notable proportion of patients with LC also have conditions such as hypertension and high cholesterol. A variety of reasons could explain this. For instance, some individuals may already have had these comorbidities before contracting COVID-19. Given that the SARS-CoV-2 virus affects multiple organ systems, including the cardiovascular system, it may have potentially exacerbated these pre-existing conditions.^10^ Conversely, some participants may have developed these conditions after COVID-19, as the immune response may remain activated for an extended prolonged period in certain patients, causing systemic inflammation that can negatively impact cardiovascular health. Considering that SARS-CoV-2 virus uses this receptor as an entry point into host cells, increased degradation of angiotensin-converting enzyme 2 (ACE2) could lead to a prolonged inflammatory cytokine storm, oxidative stress, and activation of hemostasis, all of which are characteristic indicators of severe or critical COVID-19 illness.^10,18^ Moreover, the disease and the subsequent period of isolation and recovery can induce a sedentary lifestyle and poor dietary habits, contributing to the increased risk of hypertension and high cholesterol. It is also possible that all these factors collectively influence the prevalence of these conditions in patients with LC. Based on our study, it can be theorised that patients with LC are likely to have conditions such as hypertension and high cholesterol.

Our study highlights the widespread presence of mental health issues in the LC population in northern Barcelona. Severe levels of stress, anxiety, and depression were reported in all sexes, occupational settings, and educational levels. We observed an alarmingly high prevalence of ’extremely severe’ anxiety in both sexes and at all educational levels. Similar trends were seen for depression levels reported among participants. These findings point to the significant mental health burden accompanying persistent LC symptoms. This is in line with meta-analyses showing common residual symptoms among COVID-19 survivors at 1-year post infection. These symptoms include mental health outcomes such as depression or anxiety.^3,19–21^ Also, larger studies that have found persistent anxiety and depression beyond 6–12 months after acute SARS-CoV-2 infection^22^ or up to 8 months post-infection.^23^ It is conceivable that LC may instigate the onset of mental disorders. Given the profound physiological and psychological stress that COVID-19 can inflict on individuals, a complex interplay of biological, psychological, and social factors could contribute to the development of mental health issues in the aftermath of the disease.^19^

Although the field of work and educational level showed no significant differences between sexes, these factors seem to play a role in the mental health outcomes. The significant difference in depression levels between healthcare and non-healthcare workers (*P* = 0.04) underlines the unique mental health needs and resilience of different occupational groups. The considerable prevalence of overweight individuals in both sexes could indicate a role for BMI in the LC recovery process and related mental health outcomes, which is an area worthy of further investigation.

Overall, the high level of depression, anxiety, and stress symptoms in LC in our study could be shaped by external determinants of health, especially by some of the social axes such as gender or educational levels. However, several potential biomedical mechanisms of LC pathogenesis,^10^ which may relate to mood disorders (such as depression, anxiety, and stress), are worth exploring These mechanisms range broadly from immune dysregulation and cytokine abnormalities to autoimmunity, viral persistence and reactivation, organ and vascular damage, dysfunctional neurological signalling, microbiota disruption, clotting, and endothelial abnormality.

In the following text, we will explain how potentially several mechanisms of LC pathogenesis may be related to the mood problems in patients with LC: (a) immune dysregulation as a theorised mechanism of LC pathogenesis potentially causes brain inflammation and affects brain function, potentially causing mood disorders;^24–26^ (b) the elevated cytokines may influence the functional changes in microglia, which can strongly influence neuronal network activity therefore contributing to the pathological outcomes in stress;^27–29^ (c) the potential autoantibodies targeting neurological tissues in LC could lead to brain tissue injury and chronic damage potentially leading to mood disturbances through chronic inflammation that may cause higher order of abnormal neurological signalling that might directly induce mood disorders;^26^ (d) persistent viral activity resulting in sustained inflammation may be linked to mood disorders;^25^ (e) vascular and brain organ damage may lead to reduced blood flow and affect mood regulation and cognitive function;^30^ (f) endothelial abnormalities might compromise the blood-brain barrier, allowing harmful substances to affect brain health; and (g) lastly, gut microbial imbalances (dysbiosis) associated with LC could be linked not only to digestive issues but also to disorders in remote organs such as the brain. Recent findings indicate that gut bacteria can influence the central nervous system’s function and inflammatory responses. The nervous system and the digestive system communicate via a two-way communication network known as the gut-brain axis.^31^ This network involves various connectors such as the vagus nerve, the immune response, and bacterial by-products. If patients with LC experience dysbiosis, these connectors can malfunction. This may lead to changes in the blood-brain barrier’s permeability and increased inflammation in the nervous system, allowing harmful byproducts to enter, potentially affecting brain health and leading to mood disturbances.^31^

In our sample in northern Barcelona (Spain), a high prevalence of severe stress and extremely severe anxiety was observed. Additionally, significant differences in anxiety and depression were found based on age and job role, with older individuals and non-healthcare workers showing higher relative risks (*P*<0.05). These data enable the identification of the most affected risk groups, which is essential for designing specific interventions. For example, if a high prevalence is detected among non-healthcare workers, specific psychological support programmes can be developed for this group. Knowing the prevalence justifies and guides the allocation of resources for prevention, treatment, and rehabilitation programmes, such as the implementation of cognitive-behavioral therapies for individuals with high levels of post-COVID anxiety. These data are also fundamental in influencing the formulation of more effective public health policies, allowing for the creation of mental health awareness campaigns based on local needs. Finally, the data help healthcare providers adapt their practices to the specific needs of their population, such as additional training for primary care physicians on the detection and management of depression and anxiety in patients with LC.

### Implications for research and practice

A high prevalence of depressive, anxious, and stressed symptoms has been observed among patients with LC in northern Barcelona. In particular, more than 40% of patients presenting with extremely severe anxiety is a cause for concern. These findings underscore the importance of providing prolonged care and mental health support for survivors of COVID-19 with LC. Additionally, it is crucial to identify vulnerable populations to implement targeted interventions. Future studies should consider these aspects to gain a more complete understanding of the impact of LC on mental health and serve as a starting point for the development of targeted interventions. However, further long-term research is required to delve deeper into the underlying mechanisms and evolution of mental health in people with LC. In conclusion, these findings highlight the need to identify individuals at potential risk of developing mental health problems early and to promote early access to appropriate interventions to prevent the development of mental health disorders.

## Data

The dataset relied on in this article is available from the corresponding author on reasonable request.
